# Notch signaling and proneural genes work together to control the neural building blocks for the initial scaffold in the hypothalamus

**DOI:** 10.3389/fnana.2014.00140

**Published:** 2014-12-02

**Authors:** Michelle Ware, Houda Hamdi-Rozé, Valérie Dupé

**Affiliations:** Institut de Génétique et Développement de Rennes, Faculté de Médecine, CNRS UMR6290, Université de Rennes 1Rennes, France

**Keywords:** early axon scaffold, forebrain, differentiation, tract of the postoptic commissure, mammillotegmental tract, hypothalamus patterning, ASCL1

## Abstract

The vertebrate embryonic prosencephalon gives rise to the hypothalamus, which plays essential roles in sensory information processing as well as control of physiological homeostasis and behavior. While patterning of the hypothalamus has received much attention, initial neurogenesis in the developing hypothalamus has mostly been neglected. The first differentiating progenitor cells of the hypothalamus will give rise to neurons that form the nucleus of the tract of the postoptic commissure (nTPOC) and the nucleus of the mammillotegmental tract (nMTT). The formation of these neuronal populations has to be highly controlled both spatially and temporally as these tracts will form part of the ventral longitudinal tract (VLT) and act as a scaffold for later, follower axons. This review will cumulate and summarize the existing data available describing initial neurogenesis in the vertebrate hypothalamus. It is well-known that the Notch signaling pathway through the inhibition of proneural genes is a key regulator of neurogenesis in the vertebrate central nervous system. It has only recently been proposed that loss of Notch signaling in the developing chick embryo causes an increase in the number of neurons in the hypothalamus, highlighting an early function of the Notch pathway during hypothalamus formation. Further analysis in the chick and mouse hypothalamus confirms the expression of Notch components and *Ascl1* before the appearance of the first differentiated neurons. Many newly identified proneural target genes were also found to be expressed during neuronal differentiation in the hypothalamus. Given the critical role that hypothalamic neural circuitry plays in maintaining homeostasis, it is particularly important to establish the targets downstream of this Notch/proneural network.

## Introduction

The hypothalamus is an evolutionary ancient structure in the rostral brain that plays a central role in the regulation of physiological processes such as hunger, thermoregulation, reproduction and behavior in adult vertebrates. The adult hypothalamus is subdivided into regions, each containing well documented clusters of neurons with defined functions (Simerly, 2004). Countless work involving physiological and genetic studies has focused on signaling molecules and transcription factors that control hypothalamus morphogenesis and the emergence of different neuronal subtypes (Shimogori et al., 2010). However, relatively little attention has been paid to the process through which the initial neurons are induced and specified in the primordium of the vertebrate hypothalamus, despite their key roles in pioneering the major axon pathways in the forebrain (Wilson et al., 1990; Mastick and Easter, 1996; Ware and Schubert, 2011). The first differentiating cells of the hypothalamus will give rise to neurons that form the nucleus of the tract of the postoptic commissure (nTPOC) and the nucleus of the mammillotegmental tract (nMTT). Recent advances in the chick model has established that a Notch/proneural regulatory loop is implicated very early during the differentiation of these neurons (Ratié et al., 2013). The aim of this review is to highlight a role for Notch signaling during nTPOC and nMTT differentiation; including key findings from zebrafish, chick and mouse models, which has contributed to our understanding of this field. A potential cascade involving *Ascl1* and target genes will be discussed to determine the possible regulation of these initial hypothalamic neurons.

## Patterning of the vertebrate hypothalamic primordium

During early embryogenesis the hypothalamus develops within the secondary prosencephalon (Puelles and Rubenstein, 2003; Martinez-Ferre and Martinez, 2012; Puelles et al., 2012). Developmental studies performed in zebrafish, chick and mouse indicate Sonic Hedgehog (SHH), secreted by the underlying prechordal plate mesendoderm, induces the formation of the hypothalamus (Dale et al., 1997; Mathieu et al., 2002; Aoto et al., 2009). Loss of *Shh* leads to missing ventral structures including the hypothalamus in zebrafish (Varga et al., 2001) and mouse (Chiang et al., 1996). In humans, mutations in the *Shh* gene results in holoprosencephaly, the most frequent human brain malformation that includes hypothalamic defects (Mercier et al., 2011). However, SHH alone is not sufficient to induce specific hypothalamus identity. The prechordal plate expresses numerous other secreted proteins that are involved in the development of the overlying hypothalamus primordium including Wnt antagonists, NODAL and Bone Morophogenic Proteins (BMP; Pera and Kessel, 1997; Kiecker and Niehrs, 2001; Mathieu et al., 2002; Manning et al., 2006; Cavodeassi and Houart, 2012).

Specific patterning of the hypothalamus begins when the hypothalamic primordium expresses the transcription factor *Nkx2.1* from Hamburger and Hamilton stage (HH)8 in chick and embryonic day (E)8 in mouse (Shimamura et al., 1995; Pera and Kessel, 1998; Sussel et al., 1999; Crossley et al., 2001). This expression of *Nkx2.1*, along with *Nkx2.2* is dependent on the presence of *Shh* in the prechordal plate (Barth and Wilson, 1995; Pera and Kessel, 1997; Rohr et al., 2001; Mathieu et al., 2002). SHH is then required to coordinate tissue growth and acquisition of anteroposterior (AP), dorsoventral (DV) and mediolateral patterning of the hypothalamus (Manning et al., 2006; Szabó et al., 2009).

**Figure 1:**
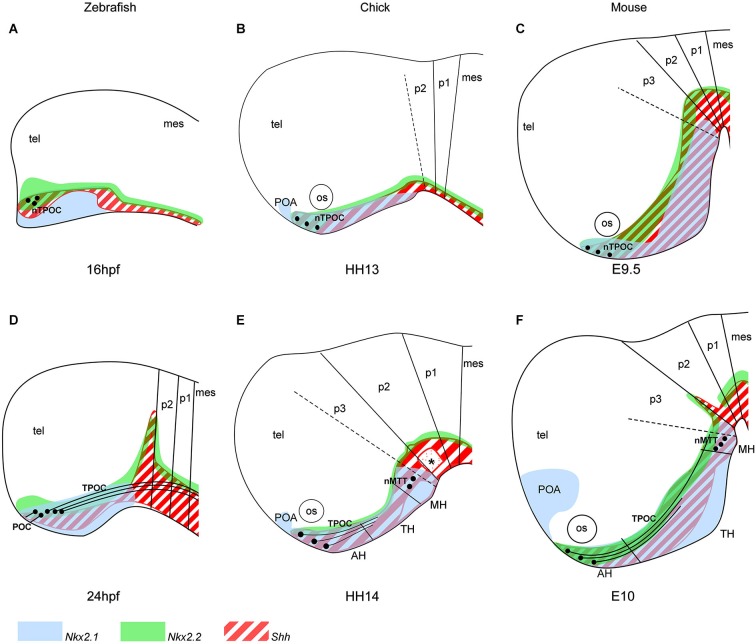
**Organization of the hypothalamic primordium in the rostral vertebrate brain. (A–C)** The first nTPOC neurons arise in the hypothalamus in zebrafish **(A)** at 16 hpf, chick **(B)** at HH13 and mouse **(C)** at E9.5. **(D)** Axons project caudally from the nTPOC forming the TPOC and rostrally from the nTPOC to form the POC in zebrafish at 24 hpf. **(E, F)** The nTPOC neurons begin projecting axons and the first nMTT neurons arise in chick at HH14 **(E)** and in mouse **(F)** at E10. The hypothalamus is specifically marked by three genes, *Nkx2.1* (light blue), *Nkx2.2* (green) and *Shh* (red stripes). **(A, D)** Gene expression in zebrafish is based on the following studies: *Shh, Nkx2.2* (Barth and Wilson, 1995; Hjorth and Key, 2001) and *Nkx2.1a* (Rohr and Concha, 2000; Rohr et al., 2001). **(B, E)** Gene expression in chick is based on the following studies: *Shh* (Bardet et al., 2010), *Nkx2.1* (Ratié et al., 2013) and *Nkx2.2* (Gimeno and Martinez, 2007). **(C, F)** Gene expression in mouse is based on the following studies: *Shh* (Shimamura et al., 1995; Alvarez-Bolado et al., 2012), *Nkx2.1* and *Nkx2.2* (Shimamura et al., 1995). **(E, F)** The three subdivisions of the hypothalamus (AH, TH and MH) in chick and mouse is based on *Shh* expression (Alvarez-Bolado et al., 2012; Ratié et al., 2013). **(E)** Asterisk, *Shh* negative region, overlapping where the ventral MLF neurons differentiate. For all schematics, subdivisions of the brain is based on the prosomeric model (Mastick and Easter, 1996; Hauptmann et al., 2002; Puelles and Rubenstein, 2003; Ware and Schubert, 2011). **(D–F)** Zona limitans intrathalamica (ZLI) marks the p2/p3 boundary. Although other neuronal populations are present in the brain at these stages, they are not added to focus on the hypothalamic neurons. AH, anterior hypothalamus; mes, mesencephalon; MH, mammillary hypothalamus; nMTT, nucleus of the tract of the mammillotegmental tract; nTPOC, nucleus of the tract of the postoptic commissure; os, optic stalk; POA, postoptic area; POC, postoptic commissure; p1–p3, prosomeres 1–3; TPOC, tract of the postoptic commissure; TH, tuberal hypothalamus; tel, telencephalon.

At HH10, *Shh, Nkx2.1* and *Nkx2.2* expression expands in the basal plate of the chick prosencephalon, with the same rostral expression at the level of the presumptive anterior hypothalamus (AH) that corresponds to the prospective chiasmatic area (also called suboptical domain) (Crossley et al., 2001). A new *Nkx2.1* expression domain develops at HH12, just rostral to the hypothalamus in the basal telencephalon called the postoptic area (POA). In zebrafish and mouse, the same dynamic expression patterns of *Shh, Nkx2.1* and *Nkx2.2* is present within the hypothalamus (Figure 1). By HH13 in chick and E9.5 in the mouse, *Shh* and *Nkx2.1* expression has expanded further and the hypothalamic primordium is morphologically evident. Studies in chick show that once the hypothalamic primordium is established, SHH down-regulation mediated by local production of BMPs is necessary for establishing region-specific transcriptional profiles (Patten and Placzek, 2002; Manning et al., 2006; Ohyama et al., 2008). This leads to the subdivisions of the primordial hypothalamus into three regions, the AH, the tuberal hypothalamus (TH) and the mammillary hypothalamus (MH), with each region expressing specific markers (Figure 1; Alvarez-Bolado et al., 2012; Wolf and Ryu, 2013).

## Initial neurogenesis in the vertebrate hypothalamus

The first neurons that differentiate in the vertebrate brain give rise to the highly conserved early axon scaffold (Chitnis and Kuwada, 1990; Wilson et al., 1990; Easter et al., 1993; Mastick and Easter, 1996; Barreiro-Iglesias et al., 2008; Ware and Schubert, 2011; Ware et al., 2014). This is an important structure for the guidance of later, follower axons allowing more complex connections to form. Predating the mature hypothalamic neuronal clusters, two small GABAergic positive populations differentiate within the hypothalamus (Figure 1; Patel et al., 1994). The first neurons in the hypothalamic primordium differentiate to give rise to the nTPOC (also termed the ventro-rostral cluster (vrc) in anamniotes) at 16 hpf in zebrafish (Figure 1A; Chitnis and Kuwada, 1990; Ross et al., 1992) and HH13 in chick (Figure 1B; Ware and Schubert, 2011). An early birth-dating study has shown that hypothalamic neurogenesis in the mouse begins at E10 (Shimada and Nakamura, 1973). However, it is well-known that the initial nTPOC neurons arise at E9.5, suggesting neurogenesis begins earlier than previously thought (Figure 1C; Easter et al., 1993; Mastick and Easter, 1996; Ricaño-Cornejo et al., 2011). From the nTPOC neurons, axons extend and project caudally within the basal plate. The tract of the postoptic commissure (TPOC) axons project into the mesencephalon where these axons form part of the ventral longitudinal tract (VLT) along with the medial longitudinal fascicle (MLF) and later the mammillotegmental tract (MTT; Ware and Schubert, 2011). The MTT forms from a second set of neurons (nMTT) that differentiate later in the caudal hypothalamus of amniotes from HH14 in chick and E10 in mouse (Figures 1E,F; Puelles et al., 1987; Easter et al., 1993; Mastick and Easter, 1996). While the presence and location of the nTPOC is conserved in all vertebrates studied, the nMTT is not present in zebrafish, at least during early development (Barreiro-Iglesias et al., 2008). Neurons do however form later in the zebrafish MH, but it is not possible to comment on the homology with the nMTT (Wolf and Ryu, 2013). The postoptic commissure (POC) forms by 24 hpf, projecting axons from the nTPOC rostrally to form a commissure across the rostral midline connecting the left and right sides of the neural tube (Figure 1D; Ross et al., 1992; Bak and Fraser, 2003). The POC is likely to form in chick and mouse at later stages but has not been studied exhaustively (Croizier et al., 2011; Ware and Schubert, 2011).

The prosomeric model and hypothalamic markers such as *Shh, Nkx2.1* and *Nkx2.2* confirms the nTPOC and nMTT neurons form within the hypothalamus (Figure 1; Hjorth and Key, 2001; Puelles and Rubenstein, 2003). The nTPOC arises just below the optic stalk at the midline of the AH area and the nMTT in the lateral edge of the caudal hypothalamus in the MH (Figure 1; Easter et al., 1993). The nTPOC neurons differentiate along the boundaries of many gene expression areas in zebrafish (Macdonald et al., 1994), however the mechanism by which these neurons differentiate has been overlooked.

Some zebrafish and mouse mutants are available where the formation of these hypothalamic axon tracts is affected. Some genes are implicated in the differentiation of the neurons such as *Six3* (Ando et al., 2005), but many studies focus on the effect of gene inactivation on axon guidance, including *Fgf8* (Shanmugalingam et al., 2000), *Pax6* (Mastick et al., 1997; Nural and Mastick, 2004), Slits and Robos (Ricaño-Cornejo et al., 2011) and *Sim1/Sim2* (Marion et al., 2005). Functionally, the TPOC is important for the guidance of other axon tracts. Ablation of the TPOC axons in the zebrafish embryo affects the patterning of the early axon scaffold (Chitnis and Kuwada, 1991). In zebrafish *Cyclops* mutants, the TPOC does not form, leading to the misguidance of the tract of the posterior commissure (TPC) axons (Patel et al., 1994). More recently, a study in the embryonic mouse has shown later hypothalamic axons from the melanin-concentrating hormone (MCH) neurons use the TPOC for guidance (Croizier et al., 2011). While the potential function of the TPOC neurons is not known, lypophilic tracing shows that the TPOC axons project into the hindbrain, although the target of these axons remains a mystery (Ware and Schubert, 2011). It is also unclear whether these neurons are still present postnatally, it could be that their sole purpose is to provide axons for guidance and then simply die after connections are made in the adult brain (Easter et al., 1993). The MTT may also function in the guidance of other tracts but this has not been studied exhaustively. In mouse, the MTT is likely to guide the mammillothalamic tract (MTH) that forms later in mouse contributing to the principle mammillary tract (Marion et al., 2005). The MTH is not known to form in zebrafish or chick. The MTT axons project to the tegmentum and are described as having a role in visceral function and processing special information in the adult human brain (Alpeeva and Makarenko, 2007; Kwon et al., 2011).

No attention has been brought to the mechanism by which the nTPOC and nMTT neurons differentiate, until 2013, when Notch components were first described as being present very early in the hypothalamus of the developing chick embryo (Ratié et al., 2013). A basic PubMed search of the key words Notch and hypothalamus generated very few publications and many of which are based in adult models or describe differentiation of late forming embryonic neurons (Chapouton et al., 2011; Aujla et al., 2013). This indicates a surprising lack of investigations surrounding neurogenesis of the initial hypothalamus neurons, when considering these early neurons have been described through-out the 1990s in different vertebrate species. As these neurons contribute to the early axon scaffold and are essential for the set-up of more complex connections, it is surely essential to understand how they differentiate and how they are specified. Finally, considering Notch along with the proneural network is a well-known signaling pathway, little is known about the implication of Notch signaling or neurogenic factors involved in the formation of the nTPOC and nMTT neurons.

## Neurogenesis and the notch/proneural network

Notch signaling is an evolutionary conserved signaling pathway involved in cell-cell communication regulating multiple processes throughout development. The Notch signaling pathway has previously been reviewed in detail, here a brief outline is described (Pierfelice et al., 2011). First identified in *Drosophila*, the Notch pathway has been confirmed to have similar roles in vertebrates (Coffman et al., 1990; Artavanis-Tsakonas and Simpson, 1991; Artavanis-Tsakonas et al., 1999). The core pathway consists of the interaction between a transmembrane Notch receptor anchored in one cell, with a transmembrane Notch ligand (Delta or Serrate/Jagged) in a neighboring cell. Upon receptor-ligand binding a series of proteolytic cleavages are triggered that releases the intracellular domain of Notch (NICD), which forms a nuclear complex with recombination signal binding protein for immunoglobulin kappa J region (RBPJ). This complex activates the transcription of target genes (Tamura et al., 1995; Fortini, 2009). The best characterized direct targets of the NICD/RBPJ complex are the Hes (Hairy-Enhancer of Split) and Hey (Hes related type) genes (Jarriault et al., 1995; Maier and Gessler, 2000). They are class-C basic helix-loop-helix (bHLH) proteins that function as transcriptional repressors and can function together as homodimers or heterodimers (Iso et al., 2003).

One function of Notch relies on lateral induction, which is defined as the process by which a ligand-expressing cell stimulates those cells nearby to upregulate ligand expression, promoting ligand propagation and coordinated cell behavior (Eddison et al., 2000). The other function of Notch is lateral inhibition, whereby a ligand-expressing cell inhibits the expression of the ligand in the neighboring cells, therefore preventing those cells from adopting the same fate and generating a patched cellular pattern (Bray, 2006). It is associated with salt-and-pepper like patterns of gene expression (Fior and Henrique, 2009). For example, these two modes of Notch pathway operation coexist during inner ear development. Each mode relies on an associated gene regulatory network (Kiernan, 2013; Neves et al., 2013). Expression and functional studies suggest that lateral induction and lateral inhibition are associated with different Notch ligands that initiate signaling (Brooker et al., 2006; Saravanamuthu et al., 2009; Petrovic et al., 2014). The association of DLL1 with lateral inhibition is a general theme during neural development (Henrique et al., 1995; Adam et al., 1998; Kageyama et al., 2010).

Notch signaling has a very well-known role in neurogenesis, controlling the balance between proliferation of neural progenitor cells (NPCs) and differentiation of NPCs into neuronal and glial cells (Campos-Ortega, 1993; Chitnis et al., 1995; de La Pompa et al., 1997; reviewed by Paridaen and Huttner, 2014). In the neuroepithelium, neuron production is mostly controlled by lateral inhibition, where a regulatory loop is formed, with proneural genes controlling the expression of Notch ligands (Bertrand et al., 2002). The ligand, DLL1, can bind and active NOTCH in neighboring cells. When the Notch signaling pathway is activated, transcriptional repressors (such as Hes or Hey genes) are expressed that prevent expression of proneural genes, inhibiting differentiation and therefore cells remain as progenitors. Cells expressing the ligand and therefore lacking Notch signaling can no longer express transcriptional repressors, leading to the upregulation of bHLH proneural transcription factors such as *Ascl1* or *Neurog1/2*. Under this Notch/proneural network the cell can exit the cell cycle and undergoes neural differentiation (Bertrand et al., 2002). This differentiation step is controlled by several classes of transcription factors that determine the identity of the neuron produced. Among them, a number of bHLH differentiation genes are switched on, such as *Nhlh1* or *NeuroD4*, followed by specific neuronal genes.

## Notch signaling in the vertebrate hypothalamus primordium

There are numerous studies investigating the expression and function of Notch components, proneural genes and downstream targets. However, as mentioned previously there is very little data describing the role of Notch signaling during the differentiation of the nTPOC and nMTT neurons. When Notch signaling is inhibited in the developing chick embryo, the number of nTPOC neurons increases, along with ectopic expression of many genes within the hypothalamus, confirming Notch has a role during hypothalamic neurogenesis at this early stage (Ratié et al., 2013). This study describes a typical neurogenic phenotype expected for the loss of Notch function, working by lateral inhibition.

**Figure 2:**
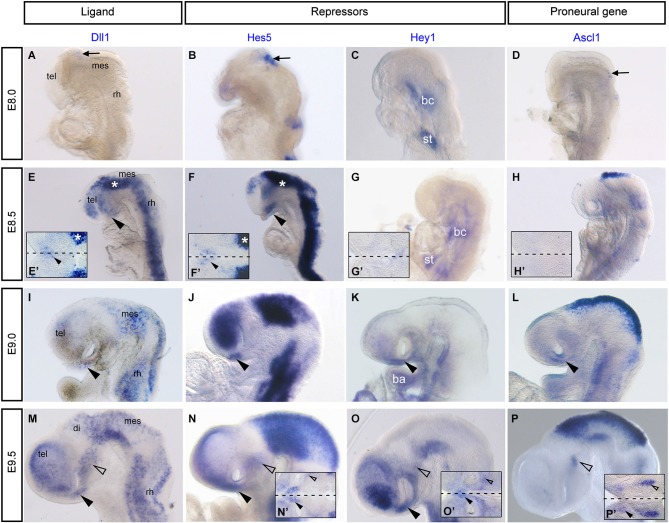
**Notch components *Dll1, Hes5, Hey1* and the proneural gene, *Ascl1* are expressed in the developing mouse hypothalamus.** Whole mount *in situ* hybridization was performed on mouse embryos as previously described (Chapman et al., 2002). Digoxigenin labeled mRNA probes were made from the following plasmids *Dll1, Hes5, Hey1* and *Ascl1* (Guillemot and Joyner, 1993). **(A–D)** E8.0, no expression of *Dll1* **(A)**, *Hes5* **(B)**, *Hey1* **(C)** and *Ascl1* **(D)** in the ventral prosencephalon. Arrows indicate dorsal expression that corresponds to NPCs and early differentiating descending tract of the mesencephalic nucleus of the trigeminal nerve (DTmesV) neurons. **(E–H)** E8.5, expression of *Dll1* **(E)** and *Hes5* **(F)** throughout the neural tube and in the AH (arrowhead). Asterisks in **(E)** and **(F)** correspond to dorsal staining in flat-mounted preparations in **(E’)** and **(F’)**. Expression of *Hey1* **(G)** and *Ascl1* **(H)** is not yet present in the AH. **(E’–H’)** Flat-mounted preparation of the hypothalamus in the same embryo at E8.5. Expression of *Dll1* **(E’)** and *Hes5* **(F’)** in the AH (arrowhead). Dashed line represents ventral midline. **(I–L)** E9.0, expression of *Dll1* **(I)**, *Hes5* **(J)** in the AH (arrowhead). Expression of *Hey1* **(K)** and *Ascl1* **(L)** begin in the AH (arrowhead). **(C, G, K)** Expression of *Hey1* in the branchial cleft (bc), septum transversum (st) and branchial arch (ba) (Leimeister et al., 1999). (M-P) E9.5, expression of *Dll1* **(M)**, *Hes5* **(N)**, *Hey1* **(O)** and *Ascl1* **(P)** in the AH (arrowhead) and in the MH (unfilled arrowhead). (**M’–P’**) Flat-mounted preparations of the hypothalamus at E9.5, arrowheads indicate expression in the AH and unfilled arrowheads indicate expression in the MH. Dashed lines represent ventral midline. mes, mesencephalon; tel, telencephalon; rh, rhombencephalon.

For the first time, the Notch components *Dll1, Hes5* and *Hey1* are shown to be expressed just before HH11 in the presumptive AH of the chick embryonic brain where the first nTPOC neurons will differentiate at HH13 (Ware and Schubert, 2011; Ratié et al., 2013). Expression of Notch components during initial neurogenesis in the zebrafish and mouse has been extensively studied, however for much of the data, it is difficult to interpret the expression in the hypothalamic primordium as no special attention was given to this area at early stages. The expression of Notch receptors in zebrafish are first described at 16 hpf in the prosencephalon and appear to overlap in the area where the nTPOC forms (Bierkamp and Campos-Ortega, 1993; Dyer et al., 2014). In mouse, while *Notch3* is ubiquitously expressed in the neuroectoderm from E8.0, *Notch2* and *Hes1* are expressed in the ventral prosencephalon from E8.5 and *Notch1* is expressed from E9.5 (Reaume et al., 1992; Williams et al., 1995; Koop et al., 1996). Remarkably, little information is present in the literature about when these genes are first expressed in the developing hypothalamus (Bettenhausen et al., 1995; de La Pompa et al., 1997; Leimeister et al., 1999; Barsi et al., 2005). Therefore, in this review, expression of Notch components are analyzed using *in situ* hybridization data to deal with this deficiency (Figure 2). *Dll1, Hes5* and *Hey1* mRNA probes are used to show the presence of Notch activity, focusing more specifically in the hypothalamus. At E8.0, *Dll1, Hes5* and *Hey1* are not expressed in the mouse presumptive hypothalamus (Figures 2A–C), it is only from E8.5, before the initial neurons differentiate that *Dll1* and *Hes5* expression is first observed (Figures 2E,E’,F,F’, arrowheads). Flat-mounted preparations of the ventral midline reveal a salt-and-pepper like pattern for these genes in the rostral hypothalamus (Figures 2E’,F’). Expression continues in the AH at E9 for *Dll1* and *Hes5* (Figures 2I,J, arrowhead), while *Hey1* expression first starts to be expressed in the same region (Figure 2K, arrowhead). At E9.5, *Dll1, Hes5* and *Hey1* are first expressed in the MH where the nMTT neurons will differentiate at E10 (Figures 2M–O, unfilled arrowhead). It is important to note that the genes analyzed here are not specific for either the nTPOC or nMTT, but are also expressed by other early developing neurons such as those in the nucleus of the mesencephalic tract of the trigeminal nerve (nmesV; Figure 2). This is not surprising as Notch is a very general pathway involved in neuron progenitor expansion (Kageyama et al., 2009). Flat-mounted preparations performed at E9.5 confirm the localized expression of *Hes5* (Figure 2N) and *Hey1* (Figure 2O) in the AH.

Bringing together the data from the literature and *in situ* hybridization of mouse embryos presented here, this highlights that Notch signaling is active very early in the AH and MH where the nTPOC and nMTT neurons will develop respectively (Mastick and Easter, 1996; Ratié et al., 2013). The data also suggests that redundancy could be strong between the direct Notch target genes as multiple transcriptional repressors such as *Hes1, Hes5* and *Hey1* are expressed in the developing hypothalamus.

Like with the expression studies described in this section, no functional data about neurogenesis in the early hypothalamus is available in zebrafish and mouse. There are several models lacking Notch signaling, which exhibit an increase in neurons throughout the embryo (de La Pompa et al., 1997; Itoh et al., 2003). For example, in the zebrafish and mouse mindbomb/Mib1 mutants, *Dll1* ubiquitination is affected and aberrant neurogenesis due to lower expression of *Hes1* and *Hes5* is observed throughout the embryo (Itoh et al., 2003; Barsi et al., 2005; Koo et al., 2005). A similar phenotype is also present in RBPj mutant mice, where Notch activity is absent (Oka et al., 1995; de La Pompa et al., 1997). As all these mutant mice display early lethality, no description is available to indicate whether neurogenesis is disturbed in the hypothalamus. Conditional loss-of-function mice lacking RBPJ, using *Nkx2.1*-Cre to specifically knock-out Notch signaling in the hypothalamus, shows that Notch signaling is essential for the differentiation of late arcuate hypothalamic neurons in the mouse from E13.5 (Aujla et al., 2013). This study did not identify a role in the initial neurons, but we would assume there would be an increase in the number of nTPOC and nMTT neurons in these mutant mice.

Many other knock-out or ectopic expression studies of Notch components describe an effect on neurogenesis throughout the embryo. The *Dll1* mutant mouse has not been well studied for a neurogenesis phenotype (Hrabe de Angelis et al., 1997; Przemeck et al., 2003). However, *Dll1* does regulate primary neurogenesis in the *Xenopus* embryo (Chitnis et al., 1995).

**Figure 3:**
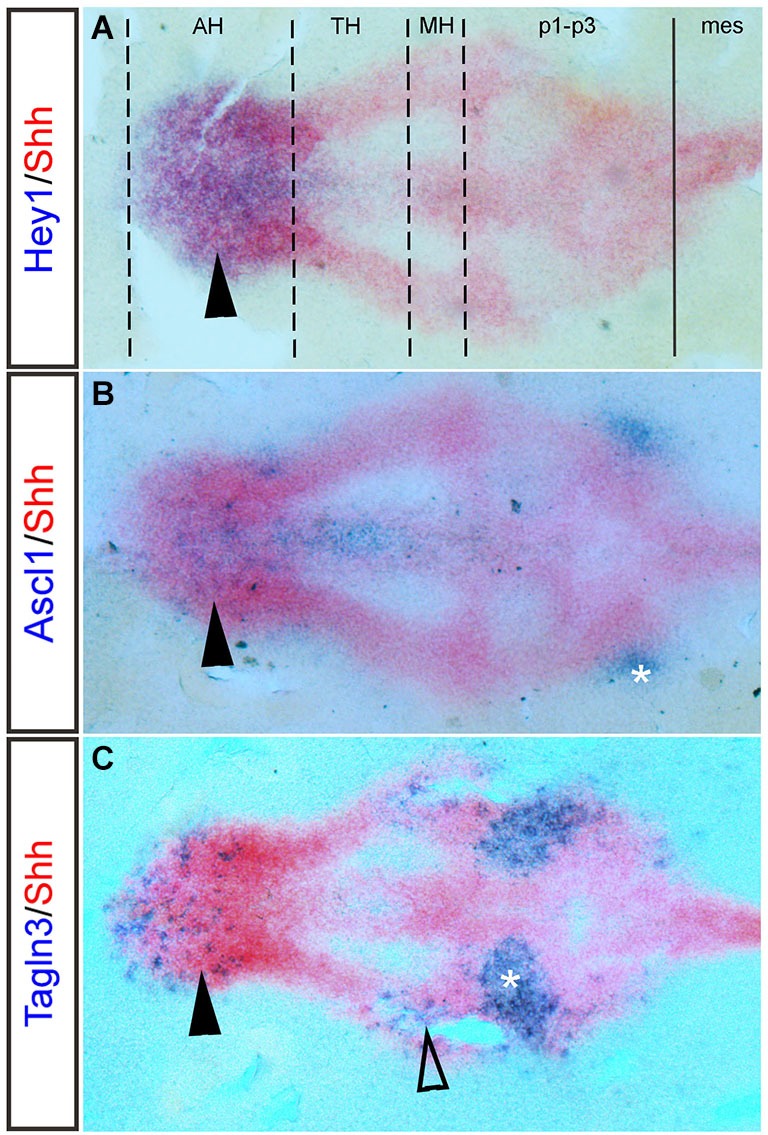
***Hey1, Ascl1* and *Tagln3* expression overlaps with *Shh* in the chick hypothalamus primordium**. Double labeling of *Hey1, Ascl1* and *Tagln3* (Purple, Digoxigenin labeled probes) at HH15 with the dynamic hypothalamic marker, *Shh* (Red, Fluorescein labeled mRNA probe) in chick confirms expression of these genes in the hypothalamic domains. **(A)** *Hey1* expression is specifically expressed in the anterior hypothalamus (AH) (arrowhead), overlapping the area where the nTPOC neurons will differentiate. Dashed lines indicate the boundaries of the hypothalamic domains, while the solid line marks the diencephalic-mesencephalic boundary (DMB). **(B)** *Ascl1* expression is located in the AH (arrowhead). Asterisk labels the nTPOC located in p1 (Ware and Schubert, 2011). **(C)** *Tagln3* expression is located in the AH (arrowhead) and in the mammillary hypothalamus (MH), overlapping where the nMTT neurons differentiate (unfilled arrowhead). Asterisk labels the ventral medial longitudinal fascicle (nMLF) located in p2 (Ware and Schubert, 2011). mes, mesencephalon; p1-p3, prosomeres 1–3; TH; tuberal hypothalamus.

There appears to be much redundancy between genes of the Notch pathway, which could explain why a function for Notch during nTPOC neuronal differentiation has not been described before in the mouse. For example, *Hes5* does not show any phenotype in single mutants (Cau et al., 2000). Double or triple knock-out mice produce more obvious phenotypes and prove redundancy occurs between these genes (Hatakeyama et al., 2004; Kageyama et al., 2008a). The absence of both *Hes1* and *Hes5* leads to aberrant neuronal localization. Interestingly, expression of *Dll1* and *Ascl1* is highly upregulated in the ventral diencephalon of E9.5 *Hes1/Hes5* double mutants as are the number of βIII-tubulin (Tuj1) positive cells (Hatakeyama et al., 2004). The capacity of these bHLH proteins to do the same job, may also explain why there is discrepancy between their expressions in chick compared with mouse. For example, flat-mounted preparations of chick embryos at HH15 (Figure 3A, arrowhead) and HH14 (Ratié et al., 2013) confirm specific expression of *Hey1* in the AH, whereas expression is throughout the developing hypothalamus in the mouse (Figures 2O,O’).

## Proneural gene expression in the vertebrate hypothalamus primordium

Induction of the Notch/proneural loop is essential in the developing hypothalamus as this will eventually lead to the correct number of cells differentiating into nTPOC and nMTT neurons as well as maintaining the progenitor population.

*Ascl1* is a well-studied proneural bHLH transcription factor, its expression and function during early embryogenesis has been well described in many vertebrates (Johnson et al., 1990; Ferreiro et al., 1993; Guillemot and Joyner, 1993; Jasoni et al., 1994; Mcnay et al., 2006). In zebrafish, *Ascl1* expression appears early at 12 hpf in cells prior to the appearance of markers indicative of overt differentiation, by 16 hpf, *Ascl1* expression overlaps with the nTPOC (Allende and Weinberg, 1994; Ando et al., 2005). In the Notch inhibited chick model, embryos display an upregulation of *Ascl1* expression in the AH, overlapping the nTPOC (Ratié et al., 2013). Flat-mounted preparations of HH15 chick hypothalamus show that *Ascl1* expression overlaps with *Shh* expression in the AH (Figure 3B, arrowhead). The expression of *Ascl1* in the hypothalamus is examined further by *in situ* hybridization between E8 and E9.5 in the mouse embryo, like with the Notch components, expression of *Ascl1* has been badly interpreted in this region (Figures 2D,H,L,P). *Ascl1* starts to be expressed in the developing hypothalamus at E9.0 (Figure 2L, arrowhead). At E9.5, flat-mounted preparations indicate that *Ascl1* is specifically expressed in a salt-and-pepper like pattern in the AH (Figures 2P,P’, arrowhead) and in the MH (Figures 2P,P’, unfilled arrowhead). *Ascl1* is important for the differentiation of late hypothalamic neurons because in *Ascl1* knock-out mice differentiation of neuroendocrine neurons is disturbed (Mcnay et al., 2006). Although the authors did not specifically look at the nTPOC or nMTT neurons it can be assumed these neurons will be affected.

During initiation of neuronal differentiation various proneural genes are recruited, but the specific proneural genes involved could be different between species and neuronal populations. Here, the expression of other proneural genes has been researched in the developing hypothalamus. Remarkably, as* Neurog1/2* are not expressed in the hypothalamus (Ratié et al., 2013), *Ascl1* appears to be the only proneural gene expressed in the ventral chick AH, at least during early development. Lateral inhibition is the process controlling differentiation of these neurons but the precise mechanisms is different between chick and mouse. There are several lines of evidence to suggest this including restriction of *Ascl1* expression to the AH in chick (Figure 2B), where in mouse *Ascl1* is expressed in both the AH and MH (Figure 2P). To date, no other proneural gene has been described in the developing chick MH. *Neurog1* and *Neurog2* are not found in the ventral hypothalamus of zebrafish and mouse (Ando et al., 2005; Mcnay et al., 2006; Osório et al., 2010), but a third member of the neurogenin family, *Neurog3* has been identified, specifically expressed in the AH (Wang et al., 2001; Villasenor et al., 2008; Pelling et al., 2011). *Neurog3* expression is regulated by *Ascl1* (Mcnay et al., 2006), but in *Neurog3* mutant mice there is no effect on early neurogenesis in the hypothalamus (Pelling et al., 2011; Anthwal et al., 2013). This lack of phenotype could be due to redundancy between the two proneural genes. It would be interesting to analyses *Ascl1/Neurog3* mutant mice to determine whether there is an additional defect in the formation of the nTPOC.

Additionally, in zebrafish and mouse, *Ascl1* and *Neurog3* may act together to control the processes of lateral inhibition leading to the differentiation of the nTPOC, whereas in chick differentiation is specifically regulated by *Ascl1*.

As the capacity to regulate differentiation steps during neurogenesis is shared by all the proneural genes (Guillemot, 2007), it may explain why neuronal differentiation in the vertebrate hypothalamus is not conserved.

## Description of proneural target genes within the hypothalamic primordium

In the absence of Notch activity during nTPOC differentiation in the chick hypothalamus, *Ascl1* is upregulated and induces expression of a wide spectrum of neuron specific genes (Castro et al., 2011; Ratié et al., 2013). While upregulation of some neuronal genes like *Nhlh1* or* Stmn2* is expected in tissue lacking Notch signaling, other genes identified are not associated with a role in hypothalamic development, such as Transgelin 3 (*Tagln3*) and Chromogranin A (*Chga*). *Nhlh1* and *Chga* mutant mice are available, but there is no phenotype or effect on neurogenesis, suggesting redundancy with other genes (Krüger and Braun, 2002; Hendy et al., 2006; Schmid et al., 2007). *Tagln3* appears to be a good marker because it is strongly expressed in the areas where both the nTPOC and nMTT form. In a flat-mounted preparation of HH15 chick hypothalamus, double labeling with *Shh* and *Tagln3* reveals expression of *Tagln3* in the AH and MH where the nTPOC and nMTT neurons are respectively located (Figure 3C, arrowhead and unfilled arrowhead). *Tagln3* is also expressed in the ventral MLF population, which are the first neurons to develop in the brain (Figure 3C, asterisk). While these target genes are all expressed in post-mitotic neurons (Theodorakis et al., 2002; Pape et al., 2008; Xie et al., 2008; Burzynski et al., 2009; Ratié et al., under review) no specific function can be attributed to these genes during nTPOC and nMTT development.

Another set of genes are specifically upregulated in the chick AH when Notch signaling is inhibited, *Slit1* and *Robo2*, which are well-known components involved in axon guidance (Chisholm and Tessier-Lavigne, 1999). They guide the TPOC axons through the hypothalamus (Devine and Key, 2008; Ricaño-Cornejo et al., 2011) and the regulation of these genes is Notch dependent (Ratié et al., 2013).

Analysis of the promoter regions in *Slit1, Robo2, Tagln3* and *Chga* reveal binding sites of *Hes5, Hey1, Ascl1* and *Nhlh1* providing further evidence these target genes are part of the Notch/proneural regulatory network involved in neuronal differentiation in the hypothalamus (Ratié et al., 2013).

## Molecular cascade of neurogenesis onset in the chick hypothalamus primordium

In order to corroborate this network of genes, the expression of Notch components and target genes is analyzed by *in situ* hybridization in the chick hypothalamus to provide further evidence for the existence of a molecular cascade that is Notch/proneural dependent.

**Figure 4:**
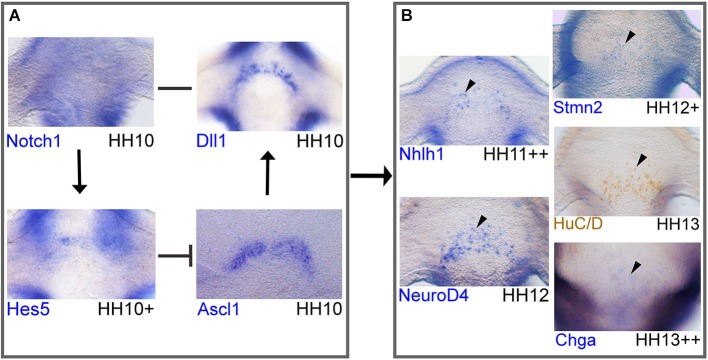
**Network of Notch/proneural genes and initial expression of downstream targets in the developing chick hypothalamus**. Whole-mount *in situ* hybridization or immunohistochemistry of markers in chick. Immunohistochemistry protocol has been described elsewhere (Lumsden and Keynes, 1989). Anti-HuC/D mouse (1:500; molecular probes; A21271) primary antibody was detected with a peroxidase-conjugated rabbit-anti-mouse secondary antibody (1:2000; Jackson ImmunoResearch; 315-035-045). Probes were obtained from cDNA and subcloned into pCRII-TOPO (Invitrogen) to make RNA probes or plasmids were obtained from other sources: *Dll1* and *Notch1* (kind gifts from Dr Frank Schubert). **(A, B)** Frontal view of the AH in the developing hypothalamus where the nTPOC neurons will differentiate. This network of genes is based on *in silico* results and data from Ratié et al., 2013. Expression of all markers, except *Notch1* have a horseshoe shape. **(A)** Notch network loop in NPCs. *Notch1* expression is ubiquitous throughout the hypothalamus at HH10. *Dll1* and *Ascl1* expression in the hypothalamus at HH10. *Hes5* expression in the hypothalamus at HH10^+^. **(B)** Genes are upregulated in post-mitotic differentiating neurons. Expression of *Nhlh1* at HH11^+^ and *NeuroD4* at HH12. *Stmn2* expression at HH12^+^, HuC/D expression at HH13 and *Chga* expression, first appears at HH13^++^ in very few cells in the developing hypothalamus. Genes are expressed in a salt-and-pepper like pattern (arrowhead). Expression confirms Notch components and *Ascl1* are expressed first, followed by the expression of downstream targets. Arrows represent activation of downstream targets. Barred lines represent repression of downstream targets. A single line represents direct binding between ligand and receptor.

The molecular cascade begins with the expression of Notch components and proneural genes followed by other bHLH transcription factors, target genes and well-known neuronal markers (Figure 4). *Notch1, Hes5, Dll1, Ascl1, Nhlh1, NeuroD4, Stmn2*, HuC/D and *Chga* are examples chosen to evaluate the stage of their first expression in the AH (Figure 4). At HH10, the first components to be expressed in the developing hypothalamus are *Notch1, Dll1* and *Ascl1*, followed by *Hes5* that form a regulatory loop (Figure 4A; Ratié et al., 2013). This mechanism has been well described in the literature for the induction of neurogenesis by lateral inhibition (Bertrand et al., 2002; Kageyama et al., 2008b).

*Notch1* is present in the AH, ubiquitously expressed (Figure 4A) compared with *Dll1, Ascl1* and *Hes5* that are expressed in a salt-and-pepper like pattern with a horseshoe shape (Figure 4). This is in agreement with a lateral inhibition model taking place in the AH. In this model, when *Ascl1* is active in a NPC, this can upregulate other bHLH genes such as *Nhlh1* (Ratié et al., 2013). *Nhlh1* and *NeuroD4* are analyzed as they are known markers of differentiation and expressed in the hypothalamus (Murdoch et al., 1999; Abu-Elmagd et al., 2001; Ratié et al., 2013). These genes are expressed from HH12, with* Nhlh1* expression appearing slightly earlier at HH11++ (Figure 4B). Other genes are upregulated from around HH13 such as, the well-known neuronal markers *Stmn2* and HuC/D but also new markers such as *Chga* (Figure 4B; Ratié et al., 2013).

BrdU labeling suggests *Dll1* expressing cells have exited the cell cycle (Henrique et al., 1995; Myat et al., 1996) therefore NPCs destined to become nTPOC neurons exit the cell cycle around HH10 as seen with *Dll1* expression (Figure 4A). It suggests that as early as HH10, the *Dll1* positive cells of the hypothalamus are destined to become neurons several stages before they become mature neurons expressing markers such as *Stmn2* or HuC/D at HH13. These results provide further evidence that the Notch/proneural loop is active in the hypothalamus from a very early stage before the first neurons appear.

## Concluding remarks

In this review, data has been discussed implicating the Notch/proneural network with a role during the differentiation of the first two groups of neurons that develop in the hypothalamus, the nTPOC and nMTT. There is still specific functional data lacking in the hypothalamus to conclude the specific mechanisms in which these neurons differentiate, but a general picture using expression data and interpretation of other functional models has been achieved. The same Notch/proneural network is likely to regulate differentiation of nTPOC neurons in zebrafish, chick and mouse. Considering the conservation of the TPOC axon tract and the Notch signaling pathway, this is not surprising. Data regarding proneural gene expression in the chick MH is still too scarce to conclude, but some of the components of the Notch/proneural network are expressed in the mouse MH before the nMTT neurons differentiate. This expression suggests the same mechanisms occur between the nTPOC and nMTT, only the players for nMTT differentiation are yet to be found in chick.

It is still not known what triggers this Notch/proneural loop in these hypothalamic NPCs. Neuronal specification during spinal cord development is initially generated by activities of two competing signaling pathways: SHH and BMP/Wnt (Ericson et al., 1997; Jessell, 2000; Liem et al., 2000). Evidence is emerging to suggest that SHH and BMP may play a similar role in the differentiation of the early hypothalamic neurons (Manning et al., 2006; Ahsan et al., 2007; Szabó et al., 2009; Alvarez-Bolado et al., 2012). However, how these signaling pathways integrate the Notch/proneural network has to be investigated in the developing hypothalamus. Future work will require a study to identify transcription factors that are necessary for the patterning of the AH and MH very early during vertebrate development.

One thing is clear, this review highlights lots of open questions regarding initial neuronal differentiation in the hypothalamus as well as general patterning of the hypothalamic regions. We hope that this review will encourage the scientific communities to investigate the phenotype of their mutants during earlier stages when the nTPOC and nMTT neurons develop.

A final thought, distinct late hypothalamic cell types dysfunction can lead to metabolic or homeostatic disorders and there is evidence that this is the case in congenital obesity (Gibson et al., 2004; Bingham et al., 2008). Therefore, could a defect in the induction and specification of the initial neurons lead to such disorders as these neurons are essential to the axon tract formation of the late hypothalamic neurons (such as the MCH neurons) (Croizier et al., 2011).

## Author contributions

Michelle Ware and Valérie Dupé set up and designed the experiments. Michelle Ware and Houda Hamdi-Rozé performed the experiments. Michelle Ware and Valérie Dupé wrote the manuscript. All authors read, discussed and edited the manuscript.

## Conflict of interest statement

The authors declare that the research was conducted in the absence of any commercial or financial relationships that could be construed as a potential conflict of interest.

## Abbreviations

AH: anterior hypothalamus
bHLH: basic helix-loop-helix
MH: mammillary hypothalamus
MTT: mammillotegmental tract
TH: tuberal hypothalamus
NPC: neural progenitor cell
nTPOC: nucleus of the tract of the postoptic commissure
nMTT: nucleus of mamillo-tegmental tract
TPOC: tract of the postoptic commissure.
